# Costs of venovenous extracorporeal membrane oxygenation (VV-ECMO) and its potential contribution to quality-adjusted life years: a mixed-methods study protocol

**DOI:** 10.1186/s12962-026-00755-8

**Published:** 2026-05-06

**Authors:** Marisa Haupert, Christian Burisch, Oliver Schöffski, Timur Sellmann

**Affiliations:** 1 Faculty of Health, Witten/Herdecke University, Witten, Germany; 2 State of North Rhine-Westphalia, Regional Government Düsseldorf, Leibniz-Gymnasium, Essen, Germany; 3 Chair of Didactics and Educational Research in Healthcare, Faculty of Health, Witten/Herdecke University, Witten, Germany; 4 School of Business, Economics and Society, Chair for Health Management, Friedrich-Alexander-University Erlangen-Nuremberg , Nuremberg, Germany; 5 Department of Anaesthesiology I, Witten/Herdecke University, Witten, Germany; 6 Department of Anesthesiology and Intensive Care Medicine, Evangelisches Krankenhaus, BETHESDA zu Duisburg, Duisburg, Germany

**Keywords:** Extracorporeal Membrane Oxygenation (MeSH term: D016638), Health Care Costs (MeSH term: D017049), Quality-Adjusted Life Years (MeSH term: D018803), Ethics, Medical (MeSH term: D004992), Treatment Outcome (MeSH term: D016896)

## Abstract

**Background:**

Venovenous extracorporeal membrane oxygenation (VV-ECMO) is a mechanical life support modality used in patients with severe respiratory failure. Despite rising case numbers worldwide, costs are substantial, while patient-centered outcomes, particularly survival and quality-adjusted life years (QALYs), remain uncertain. High complication and mortality rates also persist. Limited financial budgets, human resources, and treatment capacities in healthcare systems require critical evaluation of whether continued use of this resource-intensive intervention at the current scale is sustainable and ethically justifiable. This study aims to provide further insight into this issue and to support healthcare decision-making by offering a structured economic and ethical framework for evaluating VV-ECMO, particularly regarding resource allocation, proportionality of costs, and the justification of its use in high-cost intensive care.

**Methods:**

This mixed-methods study consists of two parts. Part A includes two systematic literature reviews. The first examines total and daily in-hospital costs per patient, as well as outcomes measured in QALYs for adult patients supported with VV-ECMO across multiple countries between 2005 and 2024. The second aim is to identify established interventions with confirmed QALY gain that can subsequently be used as comparators for VV-ECMO. Part B evaluates the results according to the principles of medical ethics: beneficence, non-maleficence, autonomy, and justice.

**Discussion:**

Given limited financial, personnel, and treatment capacities in healthcare systems, the question of how highly resource-intensive interventions such as VV-ECMO can be justified has increasing clinical, economic, and ethical relevance. By linking cost-related outcomes with a structured ethical evaluation, this study aims to contribute to a more differentiated discussion of whether and under which conditions VV-ECMO may be considered proportionate and justifiable within resource-constrained healthcare settings. Expected challenges include data availability and quality, differences in reported charges versus costs, and variation in healthcare systems, reimbursement structures, and national decision-making frameworks. These factors represent inherent limitations of international comparisons. The analysis is therefore intended as a comparative and contextual assessment rather than a formal cost-effectiveness evaluation within a single national reimbursement framework, particularly as no officially established uniform willingness-to-pay threshold per QALY exists in Germany.

**Clinical trial registration:**

Not applicable. This study does not involve interventional clinical research. The protocol was registered on the Open Science Framework (https://osf.io/24ky5).

**Supplementary Information:**

The online version contains supplementary material available at 10.1186/s12962-026-00755-8.

## Background

Venovenous extracorporeal membrane oxygenation (VV-ECMO) is an extracorporeal life support modality used in patients with severe acute respiratory failure, particularly acute respiratory distress syndrome (ARDS). It provides temporary respiratory support by draining venous blood, passing it through an external membrane oxygenator for gas exchange, and returning it to the venous circulation. This study focuses exclusively on VV-ECMO because it addresses respiratory, rather than cardiocirculatory, support.

VV-ECMO had already attained clinical and scientific attention through earlier landmark studies such as the CESAR trial [1], and this attention increased further during the COVID-19 pandemic, which provided extensive real-world data on its use. Despite high complication and mortality rates, case numbers have been increasing worldwide since its first successful application in the 1970s [2]. Randomized controlled trial evidence on VV-ECMO in severe ARDS is available from the EOLIA trial [3].

According to the Extracorporeal Life Support Organization (ELSO) Registry International Report 2022, 154,568 ECMO runs were entered into the registry from 2009 to 2022, with 780 centers contributing data overall and 557 centers submitting data in 2022 [4].

In Germany, VV-ECMO use increased by 236% from 2007 to 2018, rising from 825 to 2,768 applications, indicating a significant trend in managing severe respiratory failure [5]. For patients with ARDS the number rose by 1,143% (from 137 to 1,703) during the same period. In parallel, the proportion of VV-ECMO patients with ARDS rose from 16.6% to 61.5%, making ARDS the leading indication for VV-ECMO support [5]. A comparable increase was reported in the United States of America (USA), where ECMO use among adults rose by 433% per million hospital discharges between 2006 and 2011 [6]. However, this finding should be interpreted cautiously, as the study does not differentiate between VV-ECMO and VA-ECMO, which may differ substantially with regard to indication, duration of support, and associated resource use and costs. For Germany alone, 3397 VV-ECMO interventions in COVID-19 patients were documented between March 1, 2020, and May 31, 2021 [7].

Previous reviews have shown that the costs and resource burden of VV-ECMO are substantial but highly variable [8]. This variability makes it difficult to relate costs to potential patient outcomes. The high expenditure associated with VV-ECMO is not limited to the extracorporeal circuit itself, but also reflects the need for highly specialized personnel, prolonged intensive care, interhospital transfer logistics, and the management of severe complications[4, 8]. These factors make VV-ECMO a costly form of organ support at the individual patient level, but also a relevant institutional and systemic challenge, as its increasing use may affect staffing, ICU bed capacity, and the allocation of limited hospital resources. Comparable economic challenges have also been described for other resource-intensive forms of mechanical organ support, such as invasive mechanical ventilation, renal replacement therapy, and ventricular assist devices, where cost containment has partly been pursued through centralization of care, increasing standardization, and more selective use in appropriate patient populations.

In the USA, daily hospital costs of ECMO have been estimated at US$4,585 − 11,524 per day, with mean total hospital costs of approximately US$132,496 for patients with a respiratory indication. Personnel expenses ranged from 11% to 52% of the total amount [8]. These estimates were reported in a systematic review published in 2021 based on studies identified up to 4 March 2020 and converted to 2019 US$. In Germany total expenses of treating COVID-19 patients with ECMO have been estimated at 92,000€ per case [9].

Despite increasing ECMO use, mortality in patients receiving ECMO for respiratory indications has remained high in registry data. According to recent reports from the Extracorporeal Life Support Organization (ELSO), survival in this population remains limited, indicating that increasing case numbers have not been accompanied by a comparable improvement in patient outcomes [4].

Complications are a central factor in the evaluation of VV-ECMO because they influence both clinical outcomes and resource utilization. Registry data show that complications during ECMO support are frequent, and renal replacement therapy use is particularly common across age groups and support types. In adult respiratory ECMO, survival to hospital discharge remains limited, and a substantial proportion of survivors require transfer to long-term acute care or rehabilitation. In addition, respiratory ECMO runs are often prolonged, which may further increase exposure to complications and associated resource use [4]. At least 78% of patients experience one or more complications. Frequently reported complications include acute kidney failure, sepsis, and bleeding. However, these events may be related both to ECMO support itself and to the severity of the underlying critical illness. Regardless of causality, such complications are likely to adversely affect patient outcomes and contribute to increased healthcare costs [10].

Despite the availability of randomized controlled trial data, the overall efficacy of VV-ECMO in severe ARDS remains a matter of ongoing debate, as reflected by the findings of the EOLIA trial [3].

The high costs and substantial resource demands of VV-ECMO warrant critical evaluation, as its clinical benefit is not yet clearly established. Given the limited financial and personnel capacities of healthcare systems, it is necessary to critically evaluate whether the continued use of such a resource-intensive intervention is sustainable and ethically justifiable in its current form.

## Methods and design

The aim of this mixed-methods study is to systematically evaluate daily in-hospital costs per patient (in US$) and outcomes measured in quality-adjusted life years (QALYs) among adult patients with VV-ECMO across international settings between 2005 and 2024.

Costs will be inflated to 2025 values in the original currency using country-specific consumer price indices and subsequently converted into 2025 US$ using gross domestic product (GDP) purchasing power parity (PPP) conversion factors. All PPP and inflation data will be obtained from the Organisation for Economic Co-operation and Development (OECD).

QALYs are a standard health economic measure that combines length of life and health-related quality of life into a single outcome metric [11]. This will be conducted as a secondary data analysis based on data from federal institutions, including the Federal Statistical Office of Germany and, where applicable, the Institute for the Hospital Remuneration System, as well as published literature identified through systematic searches in PubMed/MEDLINE and other relevant bibliographic databases, and health economic evaluations. No new data will be collected, and no direct patient contact is involved.

These results will be compared with established national and international confirmed QALY-generating interventions and subsequently evaluated according to the four principles of medical ethics: beneficence, non-maleficence, autonomy and justice [12]. When comparing VV-ECMO with other interventions in terms of costs and QALY-related outcomes, the interpretation of such comparisons requires consideration of the willingness-to-pay threshold. However, such thresholds vary between countries and healthcare systems, and no universally accepted standard exists. In the German healthcare context in particular, no officially established uniform willingness-to-pay threshold per QALY is available. Therefore, comparisons with other interventions in the present study will be interpreted as an exploratory contextualization rather than as a formal cost-effectiveness assessment against a fixed national threshold.

An example of such an intervention is a BCG vaccination strategy against tuberculosis in Indonesia with estimated costs at US$112 per QALY from a healthcare perspective [13].

The present study combines critical care medicine, health economics, and medical ethics and aims to provide further insight into the comparative economic and ethical assessment and of VV-ECMO in order to inform healthcare decision-making. It will reflect VV-ECMO as an intensive care support modality from an economic and medical-ethical perspective and facilitate its contextualization within broader societal questions of resource allocation, distributive justice, and the responsible use of limited healthcare resources.

### Part A: quantitative analysis

A systematic literature search will be conducted to determine the international case numbers and total and daily direct in-hospital costs per patient of VV-ECMO (in USD) in adults. Additionally, a review is performed to identify outcomes measured in QALYs. The databases to be accessed are PubMed, Embase, Cochrane Library, CINAHL, Livivo and Scopus, as well as the economic databases National Health Service Economic Evaluation Database (NHS EED), Cost-Effectiveness Analysis Registry (CEA Registry), Institute for Clinical and Economic Review (ICER) database, and the International Health Technology Assessment Database. Search strategies have been developed using published filters from the Canadian Drug Agency search filter database [14]. When no predefined filter was available, existing filters were adapted accordingly.

The planned search strategy is detailed in the appendix (see additional file 1). The inclusion and exclusion criteria were predefined as follows: studies must have been published between 01/01/2005 and 31/12/2024, cover a study period between 2005 and 2024, be available in either German or English, and address VV-ECMO. The adult population is defined as patients aged 18 years or older. Studies will be included if they explicitly report adult VV-ECMO populations or if the study context clearly indicates an adult cohort. Studies that do not specify participant age and do not allow a clear classification as adult will be excluded. Studies focusing exclusively on VA-ECMO will be excluded. Studies including both VV- and VA-ECMO will only be included if the results for VV-ECMO are reported separately; otherwise, they will be excluded. Studies in which no distinction between ECMO configurations is made will also be excluded.

Furthermore, data collection within Germany involves reaching out to institutions such as Destatis (Federal Statistical Office of Germany) [15] and health insurance funds [16–18]. Costs are determined using the German Diagnosis-Related Groups (DRG) reimbursement catalogue for calculation.

All data will be collected for both national and international contexts. Since DRG-statistics started being documented in Germany in 2005 the timeframe will cover the years 2005–2024.

Secondary outcomes include average ECMO run duration, related complication and mortality rates.

### Statistical analysis

The cost analysis will be performed from the hospital perspective and will include direct in-hospital costs associated with VV-ECMO support, including costs related to intensive care and hospital treatment. Time series analyses will be conducted to identify trends and to assess correlations using either Kendall’s tau or Pearson’s correlation coefficient. The normality of each dataset will be assessed using the Shapiro-Wilk test. Depending on the distribution, pairwise comparisons between countries will be performed using either a t-test (for normally distributed data) or the Wilcoxon test (for non-normally distributed data). The results will then be visualized using violin plots or box plots.

### Part B: qualitative (ethical) analysis

In the second phase, a subsequent literature search will identify and evaluate other confirmed national and international QALY-generating interventions, comparing their outcomes with those from Part A, while employing the same search strategy as delineated in Part A. The planned search strategy is further detailed in the appendix, with inclusion and exclusion criteria predefined and consistent with those applied in Part A. In conclusion, the parts are evaluated according to the principles of medical ethics.

## Discussion

Healthcare systems are generally limited in financial, personnel and treatment capacities. This makes it essential to allocate existing resources efficiently and in an ethically justifiable manner. Thus, a careful consideration of costs and benefits before an intervention is obligatory. This study evaluates the comparative economic and ethical assessment of VV-ECMO.

This interdisciplinary mixed-methods study integrates quantitative and qualitative components to evaluate the research question. Expected challenges primarily relate to availability and quality of the attained data. Studies variably report costs and charges, necessitating careful differentiation when determining direct in-hospital costs. The differences between healthcare systems particularly in their financing and reimbursement may influence the results and should therefore be considered when assessing the data.

Depending on the final results, this study may contribute in different ways to future decision-making. If VV-ECMO is shown to compare favourably with other QALY-generating interventions despite its high costs, this may contribute to a more differentiated discussion of its role in resource allocation, patient selection, and institutional planning in critical care. If, however, the findings do not support the underlying hypotheses regarding economic and ethical justification, this would be equally informative, as it may help to refine future research priorities, reassess the scope of VV-ECMO use, and promote a more critical evaluation of high-cost intensive care interventions in the context of limited healthcare resources.

.

## Supplementary Information

Below is the link to the electronic supplementary material.

Supplementary Material 1

## Data Availability

The datasets used and/or analysed during the current study are available from the corresponding author on reasonable request.

## Abbreviations

ARDS: Acute Respiratory Distress Syndrome
Destatis: Federal Statistical Office of Germany
DRG: Diagnosis-Related Groups
ECMO: Extracorporeal Membrane Oxygenation
ELSO: Extracorporeal Life Support Organization
GDP: Gross Domestic Product
OECD: Organisation for Economic Co-operation and Development
PPP: Purchasing Power Parity
QALY: Quality-adjusted Life Years
USA: United States of America
VA-ECMO: Venoarterial extracorporeal membrane oxygenation
VV-ECMO: Venovenous extracorporeal membrane oxygenation
