# Supplementary material for: Costs of venovenous extracorporeal membrane oxygenation (VV-ECMO) and its potential contribution to quality-adjusted life years: a mixed-methods study protocol
Source: Cost Eff Resour Alloc. 2026 May 6;24:62. doi: 10.1186/s12962-026-00755-8 (PMC13151395; doi:10.1186/s12962-026-00755-8)
Supplement: Supplementary file 1 — Supplementary Material 1 [file 12962_2026_755_MOESM1_ESM.docx]

Planned Search Strategies

Inclusion criteria: studies have to be published between 01/01/2005 and 01/01/2025 and cover a period between 2005 and 2025, be available in either German or English and address VV-ECMO.

Excluded: only VA-ECMO or no distinction between cannulation

**Database: PubMed**

Cost

#1 ECMO

ECMO[tiab] OR "extracorporeal membrane oxygenation" [mesh] OR "extracorporeal membrane oxygenation"[tiab] OR vvecmo[tiab]

#2 Cost

"Economics"[Mesh:NoExp] OR "Costs and Cost Analysis"[mh] OR "Economics, Nursing"[mh] OR "Economics, Medical"[mh] OR "Economics, Pharmaceutical"[mh] OR "Economics, Hospital"[mh] OR "Economics, Dental"[mh] OR "Fees and Charges"[mh] OR "Budgets"[mh] OR budget*[tiab] OR economic*[tiab] OR cost[tiab] OR costs[tiab] OR costly[tiab] OR costing[tiab] OR price[tiab] OR prices[tiab] OR pricing[tiab] OR pharmacoeconomic*[tiab] OR "pharmaco-economic*"[tiab] OR expenditure[tiab] OR expenditures[tiab] OR expense[tiab] OR expenses[tiab] OR financial[tiab] OR finance[tiab] OR finances[tiab] OR financed[tiab] OR "value for money"[tiab] OR "monetary value*"[tiab] OR "models, economic"[mh] OR "economic model*"[tiab] OR "markov chains"[mh] OR markov[tiab] OR "monte carlo method"[mh] OR "monte carlo"[tiab] OR "Decision Theory"[mh] OR "decision tree*"[tiab] OR "decision analy*"[tiab] OR "decision model*“[tiab]

Taken from:

Economic Evaluations & Models - PubMed. In: Canada's Drug Agency Search Filters Database. Ottawa: Canada's Drug Agency; 2025: <https://searchfilters.cda-amc.ca/link/63>. Accessed 2025-06-17

#3 1 AND 2

QALY

#1 ECMO

ECMO[tiab] OR “extracorporeal membrane oxygenation”[mesh] OR "extracorporeal membrane oxygenation"[tiab] OR vvecmo[tiab]

#2 QALY

"Value of Life"[mh] OR "Quality of Life"[mh] OR quality of life[tiab] OR "Quality-Adjusted Life Years"[mh] OR "quality adjusted life"[tiab] OR qaly*[tiab] OR qald*[tiab] OR qale*[tiab] OR qtime*[tiab] OR "life year"[tiab] OR "life years"[tiab] OR Disability-Adjusted Life Years[mh] OR "disability adjusted life"[tiab] OR Healthy Life Expectancy[mh] OR daly*[tiab] OR disability free life expectanc*[tiab] OR haly*[tiab] OR health* life expectanc*[tiab] OR "sf36"[tiab] OR "sf 36"[tiab] OR "short form 36"[tiab] OR "shortform 36"[tiab] OR "short form36"[tiab] OR "shortform36"[tiab] OR "sf thirtysix"[tiab] OR "sfthirtysix"[tiab] OR "sfthirty six"[tiab] OR "sf thirty six"[tiab] OR "shortform thirtysix"[tiab] OR "shortform thirty six"[tiab] OR "short form thirtysix"[tiab] OR "short form thirty six"[tiab] OR "sf6"[tiab] OR "sf 6"[tiab] OR "short form 6"[tiab] OR "shortform 6"[tiab] OR "shortform6"[tiab] OR "short form6"[tiab] OR "sf6d"[tiab] OR "sf 6d"[tiab] OR "short form 6d"[tiab] OR "shortform 6d"[tiab] OR "sf six"[tiab] OR "sfsix"[tiab] OR "shortform six"[tiab] OR "short form six"[tiab] OR "sf8"[tiab] OR "sf 8"[tiab] OR "short form 8"[tiab] OR "shortform 8"[tiab] OR "shortform8"[tiab] OR "short form8"[tiab] OR "sf eight"[tiab] OR "sfeight"[tiab] OR "shortform eight"[tiab] OR "short form eight"[tiab] OR "sf12"[tiab] OR "sf 12"[tiab] OR "short form 12"[tiab] OR "shortform 12"[tiab] OR "short form12"[tiab] OR "shortform12"[tiab] OR "sf twelve"[tiab] OR "sftwelve"[tiab] OR "shortform twelve"[tiab] OR "short form twelve"[tiab] OR "sf16"[tiab] OR "sf 16"[tiab] OR "short form 16"[tiab] OR "shortform 16"[tiab] OR "short form16"[tiab] OR "shortform16"[tiab] OR "sf sixteen"[tiab] OR "sfsixteen"[tiab] OR "shortform sixteen"[tiab] OR "short form sixteen"[tiab] OR "sf20"[tiab] OR "sf 20"[tiab] OR "short form 20"[tiab] OR "shortform 20"[tiab] OR "short form20"[tiab] OR "shortform20"[tiab] OR "sf twenty"[tiab] OR "sftwenty"[tiab] OR "shortform twenty"[tiab] OR "short form twenty"[tiab] OR "hql"[tiab] OR "hqol"[tiab] OR "h qol"[tiab] OR "hrqol"[tiab] OR "hr qol"[tiab] OR "hye"[tiab] OR "hyes"[tiab] OR "healthy year equivalent*"[tiab] OR "healthy years equivalent*"[tiab] OR "pqol"[tiab] OR "qls"[tiab] OR "quality of wellbeing"[tiab] OR "quality of well being"[tiab] OR "index of wellbeing"[tiab] OR "index of well being"[tiab] OR "qwb"[tiab] OR "nottingham health profile*"[tiab] OR "sickness impact profile"[tiab] OR "health status indicators"[mh] OR "health utilit*"[tiab] OR "health status"[tiab] OR "disutilit*"[tiab] OR "rosser"[tiab] OR "willingness to pay"[tiab] OR "standard gamble*"[tiab] OR "time trade off"[tiab] OR "time tradeoff"[tiab] OR "tto"[tiab] OR "hui"[tiab] OR "hui1"[tiab] OR "hui2"[tiab] OR hui3[tiab] OR eq[tiab] OR euroqol[tiab] OR "euro qol"[tiab] OR eq5d[tiab] OR "eq 5d"[tiab] OR euroqual[tiab] OR "euro qual"[tiab] OR "duke health profile"[tiab] OR "functional status questionnaire"[tiab] OR "dartmouth coop functional health assessment*"[tiab] OR (utilit*[tiab] AND (valu*[tiab] OR measur*[tiab] OR health[tiab] OR life[tiab] OR estimat*[tiab] OR elicit*[tiab] OR disease[tiab] OR score*[tiab] OR weight[tiab])) OR (preference*[tiab] AND (valu*[tiab] OR measur*[tiab] OR health[tiab] OR life[tiab] OR estimat*[tiab] OR elicit*[tiab] OR disease[tiab] OR score*[tiab] OR instrument[tiab] OR instruments[tiab]))

Taken from
Economic - Health Utilities / Quality of Life - Standard - PubMed. In: Canada's Drug Agency Search Filters Database. Ottawa: Canada's Drug Agency; 2025: <https://searchfilters.cda-amc.ca/link/65>. Accessed 2025-06-17.

#3 1 AND 2

**Database: Embase.com**

ECMO

1. "extracorporeal oxygenation"/de

2. ECMO:ti,ab,kw

3. extracorporeal membrane oxygenation:ti,ab,kw

4. vvecmo:ti,ab,kw

5. 1 OR 2 OR 3 OR 4

Cost

6.  "Economics"/de

7. "Cost"/de

8.  "health economics"/exp

9.  "Budget"/de

10. budget*:ti,ab,kw

11. (economic* or cost or costs or costly or costing or price or prices or pricing or pharmacoeconomic* or pharmaco-economic* or expenditure or expenditures or expense or expenses or financial or finance or finances or financed):ti,kw

12. (economic* or cost or costs or costly or costing or price or prices or pricing or pharmacoeconomic* or pharmaco-economic* or expenditure or expenditures or expense or expenses or financial or finance or finances or financed):ab

13. (cost* NEAR/2 (effective* or utilit* or benefit* or minimi* or analy* or outcome or outcomes)):ab,kw

14. (value NEAR/2 (money or monetary)):ti,ab,kw

15. "Statistical Model"/de

16. "economic model"/exp

17. economic model*:ab,kw

18. "Probability"/de

19.  markov:ti,ab,kw

20. "monte carlo method"/de

21.  monte carlo:ti,ab,kw

22. "Decision Theory"/de

23. "Decision Tree"/de

24. (decision* NEAR/2 (tree* or analy* or model*)):ti,ab,kw

25. 6 OR 7 OR 8 OR 9 OR 10 OR 11 OR 12 OR 13 OR 14 OR 15 OR 16 OR 17 OR 18 OR 19 OR 20 OR 21 OR 22 OR 23 OR 24

26. 5 AND 25

QALY/HRQoL

6. "socioeconomics"/de

7. "Quality of Life"/exp

8. quality of life:ti,kw

9.  ((instrument or instruments) NEAR/3 quality of life):ab

10.  "Quality-Adjusted Life Year"/de

11. quality adjusted life:ti,ab,kw

12. (qaly* or qald* or qale* or qtime* or life year or life years):ti,ab,kw

13. "disability-adjusted life year"/de

14. disability adjusted life:ti,ab,kw

15. "healthy life expectancy"/de

16. (daly* or disability free life expectanc* or haly* or health* life expectanc*):ti,ab,kw

17. "Short form 36"/exp

18. (sf36 or sf 36 or short form 36 or shortform 36 or short form36 or shortform36 or sf thirtysix or sfthirtysix or sfthirty six or sf thirty six or shortform thirtysix or shortform thirty six or short form thirtysix or short form thirty six):ti,ab,kw

19. (sf6 or sf 6 or short form 6 or shortform 6 or sf six or sfsix or shortform six or short form six or shortform6 or short form6):ti,ab,kw

20.  (sf8 or sf 8 or sf eight or sfeight or shortform 8 or shortform 8 or shortform8 or short form8 or shortform eight or short form eight):ti,ab,kw

21. (sf12 or sf 12 or short form 12 or shortform 12 or short form12 or shortform12 or sf twelve or sftwelve or shortform twelve or short form twelve):ti,ab,kw

22. (sf16 or sf 16 or short form 16 or shortform 16 or short form16 or shortform16 or sf sixteen or sfsixteen or shortform sixteen or short form sixteen):ti,ab,kw

23. (sf20 or sf 20 or short form 20 or shortform 20 or short form20 or shortform20 or sf twenty or sftwenty or shortform twenty or short form twenty):ti,ab,kw

24. (hql or hqol or h qol or hrqol or hr qol):ti,ab,kw

25. (hye or hyes):ti,ab,kw

26. (health* NEAR/2 year* NEAR/2 equivalent*):ti,ab,kw

27.  (pqol or qls):ti,ab,kw

28.  (quality NEAR/2 wellbeing or quality NEAR/2 well being or index NEAR/2 wellbeing or index NEAR/2 well being or qwb):ti,ab,kw

29. nottingham health profile*:ti,ab,kw

30. "nottingham health profile"/de

31. sickness impact profile:ti,ab,kw

32. "sickness impact profile"/de

33. "health status indicator"/de

34. (health NEAR/3 (utilit* or status)):ti,ab,kw

35. (utilit* NEAR/3 (valu* or measur* or health or life or estimat* or elicit* or disease or score* or weight)):ti,ab,kw

36.  (preference* NEAR/3 (valu* or measur* or health or life or estimat* or elicit* or disease or score* or instrument or instruments)):ti,ab,kw

37. disutilit*:ti,ab,kw

38. rosser:ti,ab,kw

39. "Willingness To Pay"/de

40.  willingness to pay:ti,ab,kw

41.  "Standard Gamble"/de

42.  standard gamble*:ti,ab,kw

43.  "time trade-off method"/de

44.  (time trade or time tradeoff):ti,ab,kw

45.  tto:ti,ab,kw

46.  (hui or hui1 or hui2 or hui3):ti,ab,kw

47.  (eq or euroqol or euro qol or eq5d or eq 5d or euroqual or euro qual):ti,ab,kw

49.  duke health profile:ti,ab,kw

50. functional status questionnaire:ti,ab,kw

51.  dartmouth coop functional health assessment*:ti,ab,kw

52.  6 OR 7 OR 8 OR 9 OR 10 OR 11 OR 12 OR 13 OR 14 OR 15 OR 16 OR 17 OR 18 OR 19 OR 20 OR 21 OR 22 OR 23 OR 24 OR 25 OR 26 OR 27 OR 28 OR 29 OR 30 OR 31 OR 32 OR 33 OR 34 OR 35 OR 36 OR 37 OR 38 OR 39 OR 40 OR 41 OR 42 OR 43 OR 44 OR 45 OR 46 OR 47 OR 48 OR 49 OR 50 OR 51

53. 5 AND 52

Syntax adapted from:

Economic Evaluations & Models - Embase. In: Canada's Drug Agency Search Filters Database. Ottawa: Canada's Drug Agency; 2025: <https://searchfilters.cda-amc.ca/link/15>. Accessed 2025-06-17.

Economic - Health Utilities / Quality of Life - Broad - Embase. In: Canada's Drug Agency Search Filters Database. Ottawa: Canada's Drug Agency; 2025: <https://searchfilters.cda-amc.ca/link/17>. Accessed 2025-06-17.

**Database: Cochrane**

**Adapted from the PubMed Search**

Cost

#1 MeSH descriptor: [Extracorporeal Membrane Oxygenation] explode all trees

#2 ("ECMO" OR "extracorporeal membrane oxygenation" OR "vvecmo"): ti,ab,kw

#3 #1 OR #2

#4 MeSH descriptor: [Economics] this term only

#5 MeSH descriptor: [Costs and Cost Analysis] explode all trees

#6 MeSH descriptor: [Economics, Nursing] explode all trees

#7 MeSH descriptor: [Economics, Medical] explode all trees

#8 MeSH descriptor: [Economics, Pharmaceutical] explode all trees

#9 MeSH descriptor: [Economics, Hospital] explode all trees

#10 MeSH descriptor: [Economics, Dental] explode all trees

#11 MeSH descriptor: [Fees and Charges] explode all trees

#12 MeSH descriptor: [Budgets] explode all trees

#13 MeSH descriptor: [Models, Economic] explode all trees

#14 MeSH descriptor: [Markov Chains] explode all trees

#15 MeSH descriptor: [Monte Carlo Method] explode all trees

#16 MeSH descriptor: [Decision Theory] explode all trees

#17 ( budget* OR economic* OR "cost" OR "costs” OR “costly" OR "costly" OR "costing" OR "price" OR "prices" OR "pricing" OR pharmacoeconomic* OR "pharmaco-economic*" OR "expenditure" OR "expenditures" OR "expense" OR "expenses" OR "financial" OR "finance" OR "finances" OR "financed" OR "value for money" OR monetary value*) OR "economic model" OR ("arkov" OR "Monte Carlo" OR decision tree* OR decision analy* OR decision model* ):ti,ab,kw

#18 #4 OR #5 OR #6 OR #7 OR #8 OR #9 OR #10 OR #11 OR #12 OR #13 OR #14 OR #15 OR #16 OR #17 with Cochrane Library publication date Between Jan 2000 and Jan 2025

#19 #3 AND #18 with Cochrane Library publication date Between Jan 2000 and Jan 2025

QALY

#1 MeSH descriptor: [Extracorporeal Membrane Oxygenation] explode all trees

#2 ("ECMO" OR "extracorporeal membrane oxygenation" OR "vvecmo"):ti,ab,kw

#3 #1 OR #2

#4 MeSH descriptor: [Value of Life] explode all trees

#5 MeSH descriptor: [Quality of Life] explode all trees

#6 MeSH descriptor: [Quality-Adjusted Life Years] explode all trees

#7 MeSH descriptor: [Disability-Adjusted Life Years] explode all trees

#8 MeSH descriptor: [Healthy Life Expectancy] explode all trees

#9 MeSH descriptor: [Health Status Indicators] explode all trees

#10 ("quality of life" OR "quality adjusted life" OR qaly* OR qald* OR qale* OR qtime* OR "life year" OR "life years" OR "disability adjusted life" OR daly* OR (“disability free life” NEXT expectanc*) OR haly* OR (health* NEXT life NEXT expectanc*) OR "sf36" OR "sf 36" OR "short form 36" OR "shortform 36" OR "short form36" OR "shortform36" OR "sf thirtysix" OR "sfthirtysix" OR "sfthirty six" OR "sf thirty six" OR "shortform thirtysix" OR "shortform thirty six" OR "short form thirtysix" OR "short form thirty six" OR "sf6" OR "sf 6" OR "short form 6" OR "shortform 6" OR "shortform6" OR "short form6" OR
"sf6d" OR "sf 6d" OR "short form 6d" OR "shortform 6d" OR "sf six" OR "sfsix" OR "shortform six" OR "short form six" OR "sf8" OR "sf 8" OR "short form 8" OR "shortform 8" OR "shortform8" OR "short form8" OR "sf eight" OR "sfeight" OR "shortform eight" OR "short form eight" OR "sf12" OR "sf 12" OR "short form 12" OR "shortform 12" OR "short form12" OR "shortform12" OR "sf twelve" OR "sftwelve" OR "shortform twelve" OR "short form twelve" OR "sf16" OR "sf 16" OR "short form 16" OR "shortform 16" OR "short form16" OR "shortform16" OR "sf sixteen" OR "sfsixteen" OR "shortform sixteen" OR "short form sixteen" OR "sf20" OR "sf 20" OR "short form 20" OR "shortform 20" OR "short form20" OR "shortform20" OR "sf twenty" OR "sftwenty" OR "shortform twenty" OR "short form twenty" OR "hql" OR "hqol" OR "h qol" OR "hrqol" OR "hr qol" OR "hye" OR "hyes" OR ("healthy year" NEXT equivalent*) OR ("healthy years" NEXT equivalent* OR “pqol” OR “qls” OR “quality of wellbeing” OR “quality of well being” OR “index of wellbeing” OR “index of well being” OR “qwb” OR ("nottingham health" NEXT profile*) OR "sickness impact profile"):ti,ab,kw

(health NEXT utilit* OR "health status" OR disutilit* OR "rosser" OR "willingness to pay" OR standard NEXT gamble* OR “time trade off” OR “time tradeoff” OR “tto” OR “hui” OR “hui1” OR “hui2” OR “hui3” OR “eq” OR “euroqol” OR “euro qol” OR “eq5d” OR “eq 5d” OR “euroqual” OR “euro qual” OR “duke health profile” OR “functional status questionnaire” OR ("dartmouth coop functional health" NEXT assessment*);ti,ab,kw

(utilit* NEAR/3 (valu* OR measur* OR health OR life OR estimat* OR elicit* OR disease OR score* OR weight)):ti,ab,kw

(preference* NEAR/3 (valu* OR measur* OR health OR life OR estimat* OR elicit* OR disease OR score* OR instrument OR instruments)):ti,ab,kw

#11 #4 OR #5 OR #6 OR #7 OR #8 OR #9 OR #10 add the extra sets

#12 #3 AND #11 with Cochrane Library publication date Between Jan 2000 and Jan 2025

**Database: CINAHL**

#1 ECMO

MH "extracorporeal membrane oxygenation"
OR TI "extracorporeal membrane oxygenation" OR AB "extracorporeal membrane oxygenation"

OR TI "vvecmo" OR AB "vvecmo"

#2 Cost

MH "Economics" OR MH "Costs and Cost Analysis+" OR MH "Economic Aspects of Illness" OR MH "Resource Allocation+" OR MH "Economic Value of Life" OR MH "Economics, Pharmaceutical" OR MH "Economics, Dental" OR MH "Fees and Charges+" OR MH "Budgets" OR MH "Decision Trees" OR TI budget* OR TI (economic* OR cost OR costs OR costly OR costing OR price OR prices OR pricing OR pharmacoeconomic* OR "pharmaco-economic*" OR expenditure OR expenditures OR expense OR expenses OR financial OR finance OR finances OR financed ) OR TI ( cost* N2 (effective* OR utilit* OR benefit* OR minimi* OR analy* OR outcome OR outcomes) ) OR TI ( value N2 (money OR monetary) ) OR TI (markov OR monte carlo ) OR TI ( decision* N2 (tree* OR analy* OR model*) ) OR AB budget* OR AB (economic* OR cost OR costs OR costly OR costing OR price OR prices OR pricing OR pharmacoeconomic* OR "pharmaco-economic*" OR expenditure OR expenditures OR expense OR expenses OR financial OR finance OR finances OR financed ) OR AB ( cost* N2 (effective* OR utilit* OR benefit* OR minimi* OR analy* OR outcome OR outcomes) ) OR AB ( value N2 (money OR monetary) ) OR AB ( markov OR monte carlo ) OR AB ( decision* N2 (tree* OR analy* OR model*) )

Taken from: Economic Evaluations & Models - CINAHL. In: Canada's Drug Agency Search Filters Database. Ottawa: Canada's Drug Agency; 2025: <https://searchfilters.cda-amc.ca/link/62>. Accessed 2025-06-17.

#3 QALY

MH "Economic value of Life" OR MH "Quality of Life" OR TI "quality of life" OR AB "quality of life" OR AB (instrument* N3 "quality of life") OR MH "Quality-Adjusted Life Years" OR TI "quality adjusted life" OR KW "quality adjusted life" OR TI (qaly* OR qald* OR qale* OR qtime* OR "life year" OR "life years") OR AB (qaly* OR qald* OR qale* OR qtime* OR "life year" OR "life years“) OR MH "Disability-Adjusted Life Years" OR TI "disability adjusted life" OR AB "disability adjusted life" OR MH "Healthy Life Expectancy" OR TI (daly* OR "disability free life expectanc*" OR haly* OR "health* life expectanc*") OR AB (daly* OR "disability free life expectanc*" OR haly* OR "health* life expectanc*") OR TI (sf36 OR "sf 36" OR "short form 36" OR "shortform 36" OR "short form36" OR "shortform36" OR "sf thirtysix" OR "sfthirtysix" OR "sfthirty six" OR "sf thirty six" OR "shortform thirtysix" OR "shortform thirty six" OR "short form thirtysix" OR "short form thirty six") OR AB ("sf36" OR "sf 36" OR "short form 36" OR "shortform 36" OR "short form36" OR "shortform36" OR "sf thirtysix" OR "sfthirtysix" OR "sfthirty six" OR "sf thirty six" OR "shortform thirtysix" OR "shortform thirty six" OR "short form thirtysix" OR "short form thirty six") OR TI (sf6 OR "sf 6" OR "short form 6" OR "shortform 6" OR "sf six" OR sfsix OR "shortform six" OR "short form six" OR "shortform6" OR "short form6") OR AB (sf6 OR "sf 6" OR "short form 6" OR "shortform 6" OR "sf six" OR sfsix OR "shortform six" OR "short form six" OR "shortform6" OR "short form6") OR TI ("sf8" OR "sf 8" OR "sf eight" OR "sfeight" OR "shortform 8" OR "shortform8" OR "short form8" OR "shortform eight" OR "short form eight") OR AB ("sf8" OR "sf 8" OR "sf eight" OR sfeight OR "shortform 8" OR "shortform8" OR "short form8" OR "shortform eight" OR "short form eight“) OR TI ("sf12" OR "sf 12" OR "short form 12" OR "shortform 12" OR "short form12" OR "shortform12" OR "sf twelve" OR "sftwelve" OR "shortform twelve" OR "short form twelve") OR AB ("sf12" OR "sf 12" OR "short form 12" OR "shortform 12" OR "short form12" OR "shortform12" OR "sf twelve" OR "sftwelve" OR "shortform twelve" OR "short form twelve")

OR TI ("sf16" OR "sf 16" OR "short form 16" OR "shortform 16" OR "short form16" OR "shortform16" OR "sf sixteen" OR "sfsixteen" OR "shortform sixteen" OR "short form sixteen") OR AB ("sf16" OR "sf 16" OR "short form 16" OR "shortform 16" OR "short form16" OR "shortform16" OR "sf sixteen" OR sfsixteen OR "shortform sixteen" OR "short form sixteen“) OR TI ("sf20" OR "sf 20" OR "short form 20" OR "shortform 20" OR "short form20" OR "shortform20" OR "sf twenty" OR "sftwenty" OR "shortform twenty" OR "short form twenty") OR AB ("sf20" OR "sf 20" OR "short form 20" OR "shortform 20" OR "short form20" OR "shortform20" OR "sf twenty" OR "sftwenty" OR "shortform twenty" OR "short form twenty“) OR TI ("hql" OR "hqol" OR "h qol" OR "hrqol" OR "hr qol") OR AB ("hql" OR "hqol" OR "h qol" OR hrqol OR "hr qol“)) OR TI ("hye" OR "hyes") OR AB ("hye" OR "hyes") OR TI (health* N2 year* N2 equivalent*) OR AB (health* N2 year* N2 equivalent*) OR TI ("pqol" OR "qls") OR AB ("pqol" OR "qls") OR TI ("quality of wellbeing" OR "quality of well being" OR "index of wellbeing" OR "index of well being" OR qwb) OR AB ("quality of wellbeing" OR "quality of well being" OR "index of wellbeing" OR "index of well being" OR qwb) OR TI "nottingham health profile*" OR AB "nottingham health profile*“ OR TI "sickness impact profile" OR AB "sickness impact profile" OR MH "Health Status Indicators+“ OR TI ((health* N3 (utilit* OR health* N3 status)) OR AB ((health* N3 (utilit* OR health* N3 status) OR TI (utilit* N3 (valu* OR measur* OR health OR life OR estimat* OR elicit* OR disease OR score* OR weight)) OR AB (utilit* N3 (valu* OR measur* OR health OR life OR estimat* OR elicit* OR disease OR score* OR weight)) OR TI (preference* N3 (valu* OR measur* OR health OR life OR estimat* OR elicit* OR disease OR score* OR instrument* OR instruments)) OR AB (preference* N3 (valu* OR measur* OR health OR life OR estimat* OR elicit* OR disease OR score* OR instrument* OR instruments)) OR TI disutilit* OR AB disutilit* OR TI "rosser" OR AB "rosser" OR TI "willingness to pay" OR AB "willingness to pay" OR TI "standard gamble*" OR AB "standard gamble*" OR TI ("time trade off" OR "time tradeoff") OR AB ("time trade off" OR "time tradeoff") OR TI "tto" OR AB "tto" OR TI ("hui" OR "hui1" OR "hui2" OR "hui3") OR AB ("hui" OR "hui1" OR "hui2" OR "hui3") OR TI ("eq" OR "euroqol" OR "euro qol" OR "eq5d" OR "eq 5d" OR "euroqual" OR "euro qual") OR AB ("eq" OR "euroqol" OR "euro qol" OR "eq5d" OR "eq 5d" OR "euroqual" OR "euro qual“) OR TI "duke health profile" OR AB "duke health profile" OR TI "functional status questionnaire" OR AB "functional status questionnaire" OR TI "dartmouth coop functional health assessment*" OR AB "dartmouth coop functional health assessment*")

Adapted Medline filter:
Economic - Health Utilities / Quality of Life - MEDLINE. In: Canada's Drug Agency Search Filters Database. Ottawa: Canada's Drug Agency; 2025: <https://searchfilters.cda-amc.ca/link/19>. Accessed 2025-06-17.

**Database: Livivo**

#1 ("extracorporeal membrane oxygenation" OR "ECMO" OR "vvecmo“) AND ("cost" OR "costs" OR "economic" OR "resource burden" OR "charges")

#2 ("extracorporeal membrane oxygenation" OR "ECMO" OR "vvecmo") AND ("qaly" OR "qalys" OR "quality adjusted life year*" OR "hrqol" OR "health related quality of life")

#1 AND #2

**Scopus**

#1
TITLE-ABS-KEY ( "ecmo" OR "extracorporeal membrane oxygenation" OR "vvecmo" ) AND TITLE-ABS-KEY ( "budget" OR "economic" OR "cost" OR "price" OR "pharmacoeconomic" OR "pharmaco-economic" OR "expenditure" OR "expenses" OR "financial" OR "value for money" OR "monetary value" OR "economic model" OR "Markov chains" OR "Monte Carlo method" OR "Decision Theory" OR "decision tree" OR "decision analysis" OR "decision model" ) AND PUBYEAR > 1999 AND PUBYEAR < 2026 AND ( LIMIT-TO ( LANGUAGE , "English" ) OR LIMIT-TO ( LANGUAGE , "German" ) )

#2

TITLE-ABS-KEY ( "ecmo" OR "extracorporeal membrane oxygenation" OR "vvecmo" ) AND TITLE-ABS-KEY ( "quality of life" OR "quality adjusted life" OR hrqol OR qaly OR qalys OR "health utility" OR "health utilities" OR daly OR dalys OR "disability adjusted life" ) AND PUBYEAR > 1999 AND PUBYEAR < 2026 AND ( LIMIT-TO ( LANGUAGE , "English" ) OR LIMIT-TO ( LANGUAGE , "German" ) )

**University of York**: https://www.crd.york.ac.uk/CRDWeb/

(ECMO or extracorporealmembrane oxygenation or VVecmo ) FROM 2000 TO 2025

**CEA Registry**: <https://cevr.tuftsmedicalcenter.org/databases/cea-registry>

“ECMO” OR “Extracorporeal mebrane oxygenation”

**ICER**: https://icer.org

“ECMO”

**INAHATA**: <https://www.inahta.org/hta-database/>

"ECMO" OR "Extracorporeal membrane Oxygenation
